# The SMART personalised self-management system for congestive heart failure: results of a realist evaluation

**DOI:** 10.1186/s12911-014-0109-3

**Published:** 2014-11-25

**Authors:** Yvonne K Bartlett, Annette Haywood, Claire L Bentley, Jack Parker, Mark S Hawley, Gail A Mountain, Susan Mawson

**Affiliations:** Rehabilitation and Assistive Technology Research Group, School of Health and Related Research, University of Sheffield, Sheffield, UK; Public Health, School of Health and Related Research, University of Sheffield, Sheffield, UK

**Keywords:** Technology, Realist evaluation, User-centred design, Heart failure, Self-management

## Abstract

**Background:**

Technology has the potential to provide support for self-management to people with congestive heart failure (CHF). This paper describes the results of a realist evaluation of the SMART Personalised Self-Management System (PSMS) for CHF.

**Methods:**

The PSMS was used, at home, by seven people with CHF. Data describing system usage and usability as well as questionnaire and interview data were evaluated in terms of the context, mechanism and outcome hypotheses (CMOs) integral to realist evaluation.

**Results:**

The CHF PSMS improved heart failure related knowledge in those with low levels of knowledge at baseline, through providing information and quizzes. Furthermore, participants perceived the self-regulatory aspects of the CHF PSMS as being useful in encouraging daily walking. The CMOs were revised to describe the context of use, and how this influences both the mechanisms and the outcomes.

**Conclusions:**

Participants with CHF engaged with the PSMS despite some technological problems. Some positive effects on knowledge were observed as well as the potential to assist with changing physical activity behaviour. Knowledge of CHF and physical activity behaviour change are important self-management targets for CHF, and this study provides evidence to direct the further development of a technology to support these targets.

**Electronic supplementary material:**

The online version of this article (doi:10.1186/s12911-014-0109-3) contains supplementary material, which is available to authorized users.

## Background

### Congestive heart failure - prevalence, impact and current treatment

Congestive heart failure (CHF) is defined by the National Institute for Health and Clinical Excellence [NICE] as ‘a complex clinical syndrome of symptoms and signs that suggest impairment of the heart as a pump supporting physiological circulation. It is caused by structural or functional abnormalities of the heart’ p.19 [1]. One in fifteen people between the ages of 65 to 74 are affected [1]. In financial terms the annual cost of heart failure to the National Health Service (NHS) in the UK is around £716 million. Similar statistics are echoed across Europe and worldwide [2].

Current treatment of CHF incorporates both pharmacological and non-pharmacological components, and there is increasing emphasis on the value of self-management [3], including lifestyle choices such as increasing exercise [1]. Treatment recommendations encourage health care professionals to provide sufficient ‘personalised information, education, support and opportunities for discussion throughout their care to help them understand their condition and be involved in its management’ p. 9 [3]. However nurse-led self-care and self-management interventions have had variable results [4]. In recent years the interest in using technology to assist in the self-management of CHF has been growing [5,6], however the majority of this work focuses on symptom monitoring alone. While using technology to monitor symptoms and feed back to a health professional contains an element of self-management, depending on the level of feedback the patient receives, monitoring alone may not increase understanding, or change the actions of the patient. This type of symptom monitoring could be described as an extension of medical management as the medical professional is responsible for interpreting the symptoms, and suggesting further action. For technology to support self-management, the information must be fed back to the patient in a way that increases understanding and empowerment [7]. In a recent review of technology based systems targeting self-management for people with heart failure, over half of the fourteen papers identified reported significant improvements in behaviours such as daily weighing, medication management, exercise adherence, dietary adherence and stress reduction. However five of the studies did not show effects, so while the results of this review were promising, more research is needed to ascertain what makes technology based self-management effective [8].

### The CHF PSMS

In 2003 the Self-Management supported by Assistive, Rehabilitation and Telehealth technology (SMART) consortium (www.thesmartconsortium.org) was established to investigate the potential of technology to support and encourage holistic self-management of long-term conditions. The consortium aimed to move beyond the focus on symptom monitoring to incorporate aspects of rehabilitation, behavioural change and education that are deemed important to self-manage a complex long-term condition successfully. The SMART2 project targeted three conditions: CHF, chronic pain and stroke. The technology was developed according to user-centred design principles [9]. The CHF PSMS was designed to increase a) symptom awareness; b) knowledge of the condition, and c) physical activity of the user. Following development, the second phase of this research was the *in situ* deployment of the PSMS with people with CHF for one month. This paper reports the evaluation of this deployment phase.

### Realist evaluation

**Figure 1:**
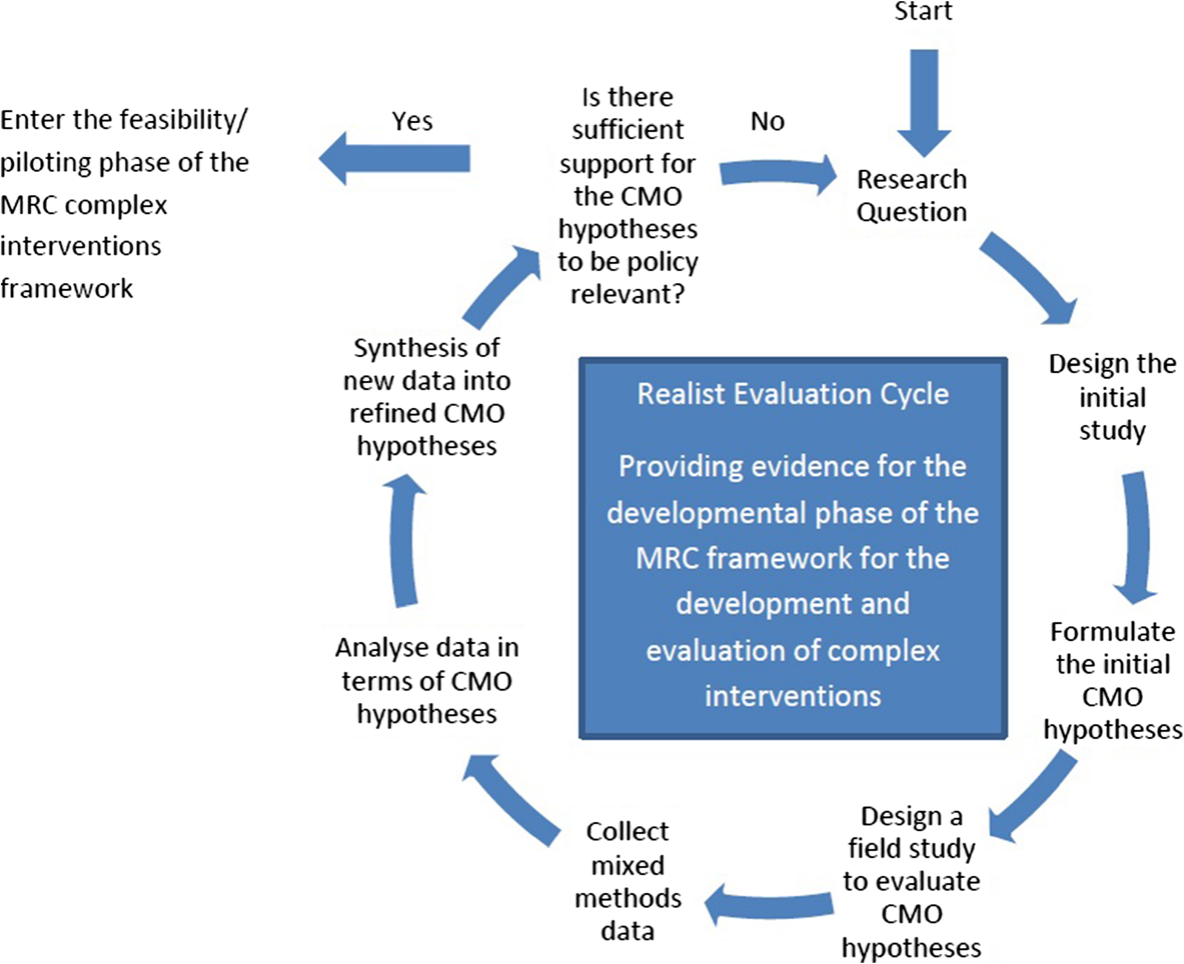
**The realist evaluation process (adapted with permission from** **[** 15 **]).**

The CHF PSMS was evaluated using a realist evaluation framework. This methodological approach involves the formulation of a number of detailed hypotheses, followed by testing their validity using multiple methods of data collection [10], see Figure 1. In this framework, the outcomes (O) that result from using an intervention result from a combination of the context (C) that it is used in, and the mechanisms (M) that form the intervention. The detailed hypotheses formed are referred to as CMO hypotheses. For example, control theory [11] has previously been applied to physical activity behaviour change, and this research has identified self-monitoring as a technique that might influence the level of physical activity performed (the outcome) [12]. Self-monitoring is therefore a theory-based technique; these are called ‘mechanisms’ in realist evaluation. An intervention designer could hypothesise that including self-monitoring (theory-based mechanism) in their intervention will increase physical activity (outcome). However, how effective this mechanism is at producing change in the outcome might also be dependent on the context the intervention is used in; for example, in the gym or at home, delivered using technology, or a paper based system. The effect on the outcome therefore is dependent on both the context and the mechanism. By formulating specific CMO hypotheses, then collecting a range of data to evaluate them, realist evaluators aim to understand the effects on outcomes and identify what works, for whom, in what circumstances [13]. Originally the realist evaluation approach was developed to evaluate health organisations (e.g. [14,15]), but has recently been adapted for use with health related interventions (including health technology interventions e.g. [16-19]). Traditional evaluation methodologies focus on whether the intervention ‘works’, but in many cases a complex intervention might work better for some people than others. Realistic evaluation allows researchers to explore this.

The CHF PSMS is a complex intervention, as defined by the Medical Research Council [20]. The framework for developing and evaluating complex interventions outlines a cycle of development, feasibility/piloting, evaluation and implementation phases [20]. Through formulating CMO hypotheses, gathering preliminary evidence of feasibility, and evaluating and revising the CMO hypotheses, realist evaluation provides evidence for the development phase of the MRC cycle by conducting what could be thought of as nested sub-cycles of the development, feasibility and evaluation stages (see Figure 1). It is an iterative process, resulting in further refinement of the hypotheses until sufficient evidence has been gathered to support the proposed CMO hypotheses and the next stage of feasibility/ piloting in the MRC framework can begin [15]. The remainder of this article describes the method followed to conduct the realist evaluation, and reports the results and their meaning in the context of self-management interventions for congestive heart failure.

## Methods

This study was granted ethical approval by Leeds (East) Research Ethics Committee (08/H1306/46)

### Development of the CHF PSMS

Initial user requirements for the CHF PSMS were identified through evidence reviews to determine best practice in self-management for each condition. This was followed by interviews and focus groups with individuals with the target conditions and health professionals to explore the lived experience and the assistance available to promote self-management. A specification for a personalised self-management system (PSMS) was produced and an initial version of the prototype was built for each of the three conditions [21]. In line with the iterative principles of user-centred design, the development phase consisted of cycles of gathering user opinion and re-design. Users commented on early paper prototypes, then low fidelity technology based prototypes, before the final version of the PSMS was presented to people with CHF in focus groups; amendments were made where necessary following each of these cycles [22].

**Figure 2:**
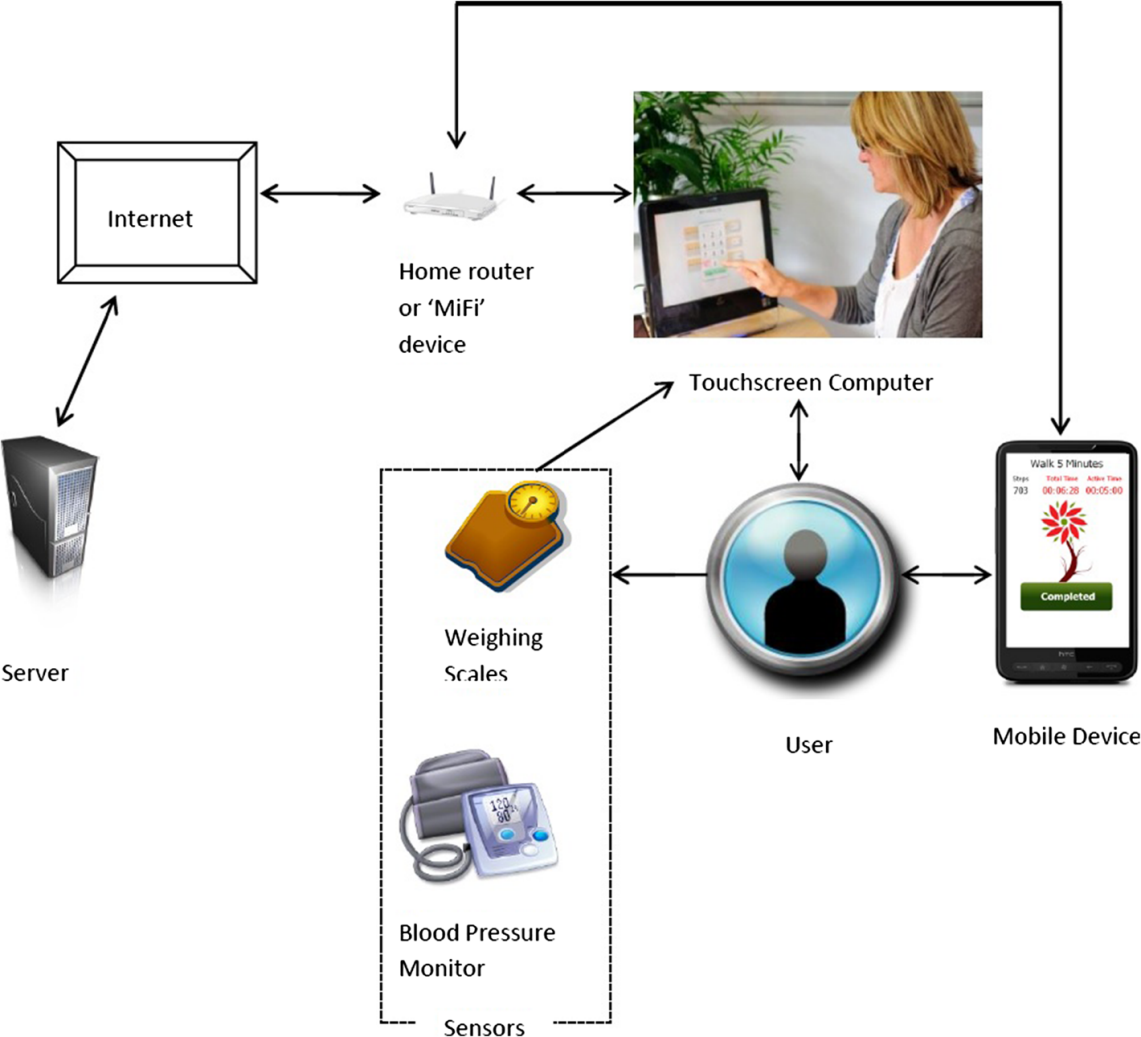
**Hardware and system structure for the CHF PSMS, mobile device, touch screen computer and sensors.**

**Figure 3:**
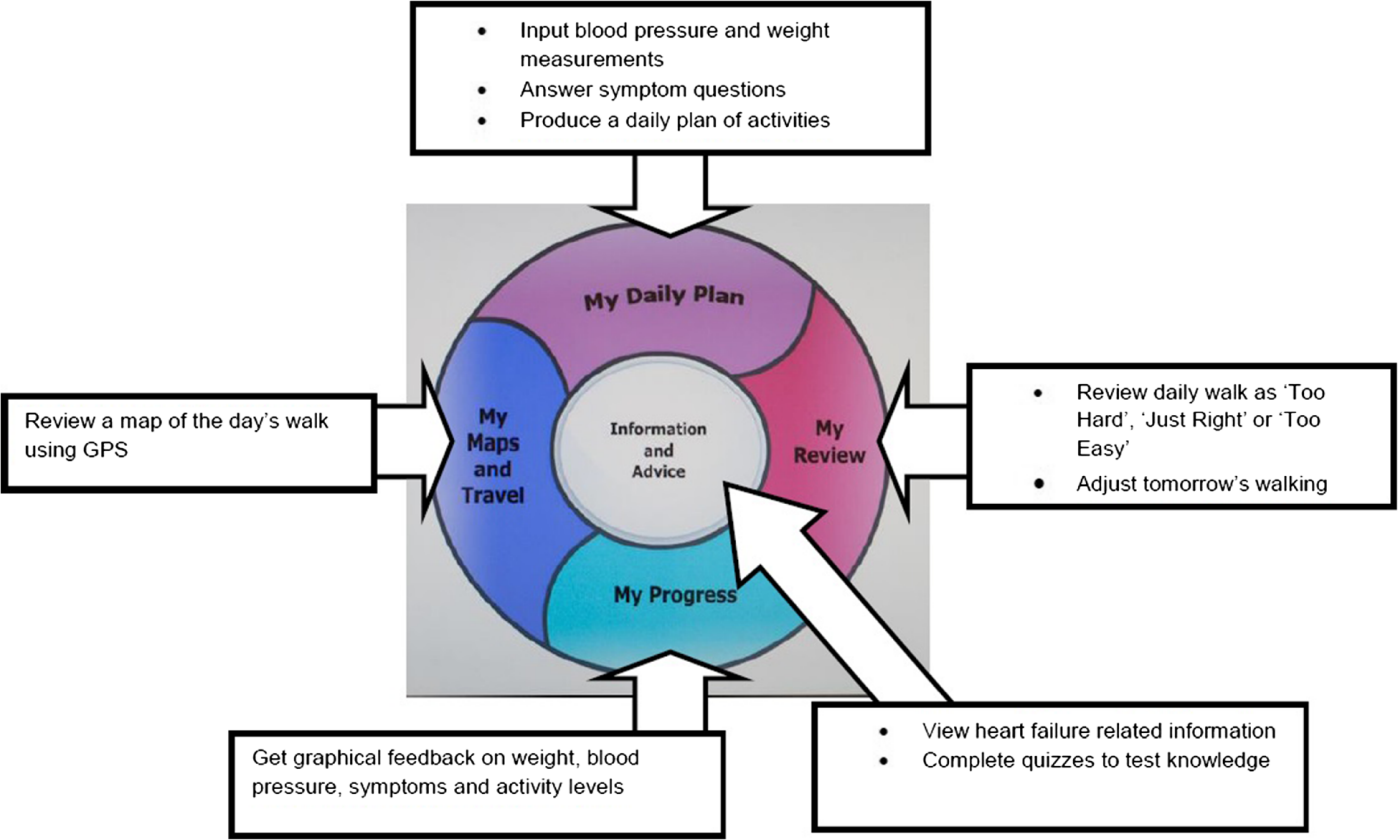
**Software components of the CHF PSMS with actions available to the user.**

Two devices were programmed to provide the necessary support; a touch screen computer (for use at home) and a touch screen mobile device (for use when out walking). Commercially available sensor devices to measure weight and blood pressure were also provided. In order to allow people without an internet connection to participate, a portable ‘MiFi’ device was provided; this allows the user to connect to the internet through a mobile phone network while at home. Together, the components of the system encouraged goal setting, enabled self-monitoring of symptoms and behaviour, encouraged the ongoing review of progress towards goals and provided access to quality-assured information about the condition (see Figures 2 and 3)

.

### Technical specifications

The touch screen computer featured a high definition (1600 × 900) multi-touch touch screen, with a dual core 1.6 GHz processor, 2 GB RAM and a 500 GB hard disk. To facilitate a positive user experience a graphical user interface (GUI) was developed using Adobe Flash Drive. The logic and computational tasks were then developed using the Java programming platform (JDK 1.6). The mobile device was a HTC HD2 phone; this model features a 480 × 800 Wide Video Graphics Array capacitive touch screen, a IGHz processor, 448 MB RAM and an internal accelerometer. The mobile application software was developed in C# using the .NET Compact Framework 3.5 to run on the Windows Mobile 6.5 Professional platform used by the HD2. An Apache Tomcat web server, and a MySQL database server were used to i) collect the information from the mobile device, and the sensors ii) decode the information and calculate the step count, weight and blood pressure and iii) store this information within the database so that it could be retrieved. Communication between the devices, as well as between the server and the devices, was essential to ensure up to date feedback could be given to the user. Communication between the devices was managed using TCP/IP socket technology combined with a customised XML protocol to pass messages, programmed using JDK 1.6. Communication between the touchscreen computer and the server, and the mobile device and the server, was managed through the participant’s home internet connection, or the ‘MiFi’ device (see Figure 2 for an outline of the system structure and Davies et al., [21] for further technical detail). As this is a proof of concept study the software is not yet available requiring further development and research.

### Defining the CMOs

The theory-based mechanisms for each CMO hypothesis were generated from literature reviews to identify theories and approaches that might inform a technology-based intervention for CHF self-management. The theories identified were from a range of discipline-specific literature including human computer interaction, cardiac rehabilitation and health psychology. For example, as described earlier, self-monitoring as a mechanism for increasing physical activity is drawn from control theory [11]. Following identification of potential theory based mechanisms from the literature, people with CHF and health care professionals took part in interviews and focus groups; this enabled the researchers to gather knowledge of people’s experiences of living with the condition, and insight from professionals who have an understanding of how a system like the PSMS might work in practice. This context-specific information was then used to further refine the CMO hypotheses (the CMOs that were established through previous research are presented in Table 1). Further detail regarding the development of the CMOs for the CHF version of the PSMS has been reported elsewhere [23]. In accord with the realist evaluation framework, these hypotheses were tested and new CMO hypotheses generated to gain an understanding of not just whether the PSMS works, but for whom, in what circumstances and how.

**Table 1:** **Hypotheses defined in terms of context, mechanisms and outcomes (CMOs) with related sources and findings**

**Context**	**Findings related to context**	**Mechanisms**	**Findings related to mechanisms**	**Outcomes**	**Findings related to outcomes**
C1. Limited access to technical support during deployment of the system	Telephone support on weekdays, potential for face to face visits	M1. Systems that have technical problems can result in low usability, and poor engagement. Therefore the system will be glitch free and fully functioning.	There were technical problems and usability was reduced.	O1. Engagement with system. Data sources: Interview; system data; System Usability Scale.	Low SUS scores, interview data showed users were engaged with the content.
C2. Differing levels of computer literacy amongst users	All participants had high levels of computer literacy	M2. User-centred design process undertaken to identify a touch screen system with simple instructions designed to be operated by those with little or no computer knowledge	Could not be tested	O2. All are able to use the system and continue to use it for the duration of the evaluation. Data sources: Interview; System Usability Scale.	This was supported, but all participants had high computer literacy.
C3. Over exertion on days when users are feeling well can result in a negative impact on subsequent days (the ‘over activity/ rest cycle’)	Not supported, at this stage	M3. Pacing is taught by the system by providing feedback on activity, and showing users weekly plans, highlighting instances of over activity.	Activity planner was not used as intended. Following initial set up participants did not keep it up to date resulting in an inaccurate record	O3. Balance between activity and rest. Data sources: Interview; system data	Not supported, further research needed to investigate context and potential mechanisms
C4. Loss of fitness and sedentary lifestyle of users resulting in fewer hobbies and interests	Not supported with the post-deployment interviews, but had been mentioned in CMO development interviews and focus groups	M4. Walking intervention to increase physical fitness	Most people reported completing the walking intervention, although not all walks were recorded by the system	O4. Ability to walk further. Data sources: Walking data, interview	Some reported improvements, objective data was unavailable due to technical problems.
C5. Lack of recognition in users of worsening condition resulting in exacerbations of symptoms and potential for admission to hospital	Not supported, participants were stable and had good awareness of symptoms that could lead to exacerbation	M5. Increasing awareness of blood pressure, weight and symptoms through self-monitoring and tailored feedback provision	Some participants reported this in the interviews	O5. Improved symptom control thus reducing need for health professional involvement. Data sources: Interview; system data	Could not be objectively tested. One person reported going to the Dr as a result of high blood pressure readings.
C6. Lack of knowledge as user is left alone to self-manage when their heart failure is stable, resulting in fewer opportunities for the health care professional to educate patients.	Although participants acknowledged they self-managed their heart failure, it was felt that as they were stable, this was appropriate, most participants had high levels of heart failure related knowledge	M6. Information and advice section contains educational material and quizzes, feedback from this and other sections should increase awareness.	Information was looked at and quizzes completed pre and post-deployment	O6. Increased levels of knowledge about self management. Data sources: Interview; Knowledge of Heart Failure questionnaire (TELER method).	Significant increase in knowledge between pre and post-deployment quizzes for those with low levels initially
C7. Self-management of heart failure involves engagement with a variety of lifestyle changes, e.g., adhering to a medication regime, restrictions to diet, monitoring weight and taking regular exercise.	Not challenged by participants. Interview data suggests participants felt behaviour change was important.	M7. The SMART2 system incorporates the following behaviour change techniques: 1. Self-monitoring of symptoms; 2. setting and reviewing goals related to user’s lifestyle; 3. providing regular feedback on performance.	Some problems with self-monitoring. Goal-setting generally supported, feedback not always attended to. Interview data reported that the system did increase walking behaviour.	O7. Behaviour change that is sustainable over the long term. Data source: Interview.	Could not be objectively tested. Interview data suggested participants perceived this as possible, if current problems were addressed.

### Deployment of the CHF PSMS

In order to test the CMOs and generate revised hypotheses, the PSMS was deployed in the homes of seven people with CHF and data were collected during the 30 day deployment period. The data collected were:

- Tracking data from system use, for example the screens that were viewed and how long each was viewed for.
- Use of the weight and blood pressure monitors; which were used and how often;
- System usability scale scores following the 30 day deployment [24,25]
- Scores from a TELER® quiz type indicator devised for the study to assess changes in CHF related knowledge. The indicator was given to participants by the researcher and completed on paper pre- and post-deployment
- Activity levels, recorded through the mobile device
- Interview data from qualitative interviews with each user collected post-deployment

See Table 1 for how these data sources relate to the CMOs. Interviews were transcribed verbatim, coded and analysed thematically under context, mechanism and outcome groupings to allow the authors to determine whether the evidence supported or negated the proposed CMOs and whether there was evidence suggesting a revised CMO.

### The TELER® method

Measuring changes in knowledge level was a key outcome for the CHF PSMS, however quantifying knowledge and ensuring that the change observed is neither random nor the result of measurement error is challenging, especially with a small sample size. The research team decided to utilise the TELER® method devised by le Roux [26,27]. As this is likely to be a novel methodology to many, it will be explained in some detail here. Within the TELER approach there are three types of outcome indicators: the functional indicator, the component indicator and the quiz style indicator, the latter measuring changes in knowledge.

As there were no quiz style indicators for CHF in the TELER® library, A.H. identified a set of statements from the heart failure related information provided by the British Heart Foundation website that could be used to assess condition specific knowledge. The statements were facts about heart failure such as ‘A common symptom of heart failure is shortness of breath’ (see Additional file 1: Appendix 2 for all quiz items) [27]. The statements had three response options, ‘agree’ , ‘disagree’ and ‘don’t know’. In good questionnaire design, where knowledge is being evaluated, half the statements need to be framed so that a positive response is correct, and half vice versa, thus prompting the respondents to think more carefully about their responses, so minimising bias or guessing [28]. The research team worked with le Roux to develop the quiz, the quiz style indicator and to conduct the multi nominal distribution function analysis [29].

The multi nominal distribution function calculates the probability of obtaining a particular response profile by chance (guessing or random event) and therefore allows the researcher to classify the respondent’s true level of knowledge. The function and component indicators in TELER are defined in terms of clinical knowledge, however the codes in the quiz style indicator are defined in terms of a probability distribution, utilising the multi nominal distribution function. This allows the analysis of the respondent’s profiles in terms of different levels of probability - for example code 1 and code 5 each represents a probability of 0.025, code 2 and 4 represent a probability of 0.14, code 3 represents a probability of 0.67 (one SD either side of the mean on a normal distribution curve). Hence a code of 1 or 5 means a statistically significant level of either no knowledge or knowledge, code 2 and 4 is acceptable knowledge with some guessing and a code of 3 too much guessing (chance or random) to identify the level of knowledge. Table 2 defines the TELER indicator code, in terms of a range of response profiles, the probability that a response profile in the range is a random event and hence the corresponding level of knowledge.

**Table 2:** **TELER® quiz style indicator for heart failure knowledge**

**TELER code**	**Scores (incorrect responses)**	**Probability**	**Level of knowledge**
0	Not assessed	None	Not assessed
1	14-15	p ≤0.025	Very poor knowledge
2	12-13	0.025 < p ≤0.16522	Moderately poor knowledge
3	9-11	0.165 < p ≤0.67033	Knowledge indeterminate
4	7-8	0.025 < p ≤0.16544	Knowledge moderately good
5	0-6	p ≤0.025	Knowledge very good

The unique nature of the TELER method allows the researchers to quantify the level of knowledge as a potential outcome from using the CHF PSMS. Underpinned by measurement theory, TELER does not add the number of responses, as do many questionnaires, but rather enables the development of a code with a unique meaning. Furthermore, the TELER method enables the researcher to quantify the level of knowledge of an individual respondent. Traditionally the total score provided by other questionnaires is taken as the respondent’s level of knowledge, however this is an invalid measure as the researcher would not know to what extent the score is a random event and therefore a valid measurement.

### Procedure

Two participants who had been involved in earlier stages of the project agreed to use the PSMS at home and in their immediate community. A further twelve potential new participants who were not involved in the development of the CMOs were identified via specialist nursing services and five of these agreed to participate. Written informed consent was provided by all participants. The seven participants were each given a training session in their own homes, where the equipment was set up and the different components of the software were explained. Participants were all left with an instruction booklet including contact details for the research team and descriptions of each component of the system. Participants were asked to use the system on a daily basis in their own homes for a four week period. When the equipment was collected at the end of this period participants were interviewed qualitatively about their experiences (see Additional file 1: Appendix 1 for topic guide).

## Results

The results are summarised in Table 3. CMOs were rejected if all three components (context (C), mechanism (M) and outcome (O)) were not supported by the data collected during the deployment phase (either through no evidence, or evidence contradictory to the hypotheses). If some components were supported, the CMO hypotheses were revised. Where the data suggested new hypotheses, new CMOs were developed. The suggested revisions and new CMOs developed during the deployment phase are presented in Table 3. The numbers in the text below indicate which of the original CMOs is being referred to e.g. C1 is the context ‘Limited access to technical support during deployment of the system’ from CMO1 in Table 1.

**Table 3:** **Results of the realist evaluation**

**Context**	**Mechanisms**	**Outcomes**
*New CMOs*		
The CHF PSMS is used in a home-setting	Hardware is acceptable to people in their homes, and fits with their everyday life	Engagement and happiness to use the system
People with CHF may have co-morbidities that will affect PSMS use	Increased pain or discomfort while walking	Participant won’t complete the recommended walk, no improvement and perhaps decline in physical activity or increase in weight.
*Revised CMOs*		
**C1. Access to technical support throughout the project**	**M1. System had technical problems, but participants used the aspects they could, and called for technical support as they needed it.**	O1a. Poor SUS score
		**O1b. Continued engagement with system**.
**C2. High level of computer literacy**	M2. User-centred design process undertaken to identify a touch screen system with simple instructions designed to be operated by those with little or no computer knowledge	O2. All are able to use the system and continue to use it for the duration of the evaluation.
**C4. Relatively active individuals have goals to lose weight and get more active**	M4. Walking intervention encouraged goal setting and increased activity	**O4. Goals were set and met, this was perceived to be useful. Weight was lost by some participants.**
**C5. Stable individuals can benefit from monitoring symptoms**	M5. Increasing awareness of blood pressure, weight and symptoms through self-monitoring and tailored feedback provision	**O5. Self-reported increased awareness of symptoms, this resulted in increased health professional involvement in one case, and medications were reviewed**
**C6. For people with low levels of heart failure knowledge**	M6. Information and advice section contains educational material and quizzes, feedback from this and other sections should increase awareness.	O6. Increased levels of knowledge about self-management. Data sources: Interview; Knowledge of Heart Failure questionnaire (TELER© method).
C7. Self-management of heart failure involves engagement with a variety of lifestyle changes, e.g., adhering to a medication regime, restrictions to diet, monitoring weight and taking regular exercise.	**M7. The SMART2 system incorporates the following behaviour change techniques: 1. Self-monitoring of symptoms; 2. setting and reviewing goals related to user’s lifestyle; 3. providing regular feedback on performance. These features must be fully functioning**	**O7. Behaviour change may be sustainable over the long term**
*Rejected CMO*		
C3. Over exertion on days when users are feeling well can result in a negative impact on subsequent days (the ‘over activity/ rest cycle)	M3. Pacing is taught by the system by providing feedback on activity, and showing users weekly plans, highlighting instances of over activity.	O3. Balance between activity and rest. Data sources: Interview; system data

Bold text identifies the components of the CMOs that have been revised.

### Contextual factors

The hypothesised context of use for the CHF PSMS was made up of both personal and environmental factors. The hypothesised environment of use for the PSMS includes the level of technical support provided (C1), and the importance of behaviour changes for appropriate self-management of heart failure (C7). As the PSMS would be used in people’s own homes, it must be accepted into people’s everyday lives - this environmental context was not included in the original CMOs, but was identified in the deployment phase. The size and appearance of the touch-screen computer in the home, as well as the portability of the mobile device, were identified as important within the context of home use:

*‘I think it’s too large. I think it’s too big. We’ve got a fairly big house and I think it was a bit obtrusive’. (CHF 102 P2, L88)*

*‘It’s another thing. You’ve got your own telephone and then it’s another thing you’ve got to have another pocket for’ (CHF 106, P2, L62)*

This context could influence all the other outcomes identified as, if people are unwilling to have the system in their homes, or in their pockets, none of the mechanisms within the intervention would have any opportunity to influence the outcomes.

While the hypothesised environmental contexts identified in the CMO development phase were corroborated, there was mixed support for the hypothesised personal contexts. For example, a range of levels of computer literacy (C2) and variance in ability to recognise symptoms were identified in the development phase as being expected across the population of people with CHF but the interviews revealed that all participants recruited for the deployment had high levels of computer literacy, indicating these factors were not part of the personal context of use for these participants:

*‘I’ve got a couple of laptops. I’ve used them and been on the net for a few years now, so I’m pretty conversant with it’ (CHF102)*

The majority of participants had been living with their condition for a number of years, and had been identified by the heart failure nurse as ‘stable’. This meant these participants had a lower chance of experiencing an exacerbation

*‘The question where you said ‘How is your breathing?’ I just found that boring because I was putting the same thing down every day’ (CHF103)*

The over activity/rest cycle (C3) and the loss of fitness in people with CHF (C4) were identified as important contextual factors when developing the CMO hypotheses. The over activity/rest cycle, however, was not identified as an issue for these participants:

*‘Well I could see that every day there was a pattern, pretty much walking the dog every day and going for a walk every day, working Monday to Friday, gym Monday, Wednesday, Friday and swimming Tuesday and Thursday. Some Saturdays I might have football on’ (CHF107)*

Changes to levels of physical activity were recognised as important in the initial stages of CHF (C4) but the majority of these participants were active currently:

*‘When you are first getting over your heart failure, the only thing you can do is potter about and walk for a bit… If you just sit there and do nothing you won’t get better anyway, actually walking and exercising is part of your medication and getting you better’ (CHF107)*

However, when asked to identify a goal they would like to achieve through the deployment phase, all seven participants reported that they would like to either lose weight, or improve or regain fitness, indicating perceived reduced levels of activity (C4).

All participants recognised that they were expected to self-manage without a great deal of health professional support when they were stable (C6), and felt this was appropriate. They indicated, however, that greater support was needed when people were less stable:

*‘I don’t need heavy involvement. The good thing for me is that I am stable, there is a lot of people that aren’t and when you aren’t stable that’s when you do seem to need a lot more advice and back up’ (CHF102)*

In conclusion, the environmental contexts identified in the development stage were corroborated during the deployment, as were three of the personal contexts, however two of the personal contexts were not relevant for these participants (C3 & C5).

### Mechanisms and outcomes

The system was designed to be used with limited technical support (C1) and, in order for users to be engaged with the system (O1), it needed to be reliable and usable (M1). Technical problems meant that this mechanism was not supported. The problems related to the mobile device connecting to the home hub:

*‘We had the trouble with the phone not connecting to the hub. The phone wasn’t registering’. (CHF109)*

The battery life of the mobile device:

*‘I also noticed that it used to lose its charge very quickly, I charged it all night but then it lost its charge very quickly’ (CHF108)*

The phone skipping to unexpected screens:

*‘It’s just in your pocket so the thing’s just there and quite often it can skip on to another programme in there’ (CHF106)*

These technical problems had the expected effects on usability outcomes (O1), with a below average usability score being given on the SUS in 5/7 cases (see Table 4). The interview data also suggested that people were frustrated with the technical problems. However engagement with the system was better than expected in light of these problems. Blood pressure was measured on 84% of the possible days across the sample; and weight on 88% of the days. Daily walks were less frequently recorded, on only 51% of the possible days. However, the interview data reports higher engagement with the walking than this, so this could have been due to the technical issues with connectivity between the mobile device and the touch screen computer; i.e. walks that were completed may not have been recorded. These high symptom monitoring readings indicate that people were happy to measure their blood pressure regularly. However, due to the existing high level of participants’ confidence and the stability of their conditions, it is not clear whether monitoring increased awareness, or resulted in fewer exacerbations (M5, O5). Participants differed in their reactions to the information received; two participants reported fluctuating blood pressure, and whilst one contacted their GP to review their medications, the other did not as they did not feel unwell.

**Table 4:** **System usability scale** [25]

**ID.**	**System usability scale score** ^**1**^	
102	75	Usability score above average
103	30	Below average
104	60	Below average
105	52.5	Below average
106	47.50	Below average
107	47.50	Below average
108	77.5	Usability score above average

^1^A score of 68 is considered average.

It was proposed that performing a daily walk while using the system (M4) would improve perceived physical fitness amongst participants (O4). As explained above, a reliable figure for the number of walks completed could not be obtained from the PSMS. Interview data suggests that people sometimes had trouble completing the walk, especially if they had other commitments, or if the weather was unsuitable:

*‘It has to fit with your lifestyle. Last Thursday we had a dinner, so had to do a lot of prep work… I helped with that so to break off from that just to go for a walk is impractical. So you can’t go for a walk every day, you have to go for a walk as it fits in with your life’ (CHF103)*

Although only one participant perceived that the intervention did have an impact on his physical activity, four out of seven reported weight loss during the deployment phase. One participant reported beginning to walk more during the intervention, but that this exacerbated the arthritis in his knees, therefore having a detrimental effect on his health. This suggests that co-morbid conditions should be considered as a contextual factor as they could have an effect on how the mechanism operates, and therefore, the outcomes of the intervention.

Relevant information was provided through the system (M6) to increase participants’ knowledge about heart failure (O6). This outcome was assessed by testing knowledge with a 15 item quiz both pre and post-deployment of the system (see Additional file 1: Appendix 2) and calculating whether there had been a change in knowledge. The TELER© analysis showed that 4/7 participants achieved a ‘very positive’ score for knowledge about heart failure in the pre-deployment quiz. The number of participants with a ‘very positive’ knowledge score following the intervention was 7/7, indicating a statistically significant improvement in knowledge during the intervention in those who had lower knowledge scores to begin with. The statements identified as inhibitory statements (indicating lower knowledge) were around appropriate diet and fluid intake. These findings indicate that CMO6 is supported, in cases where the initial level of knowledge is lower (C6).

It was proposed that, through self-monitoring, goal-setting and receiving feedback (M7), participants could introduce long-term behaviour changes (O7). The idea of self-monitoring motivating behaviour change was supported by participants:

*‘I do think it gives you a motivation. That machine’s in the corner and if you’re not doing it one particular day it monitors you. It’s down there, you can’t go backwards or scrub it out or cheat it’. (CHF106)*

However, some felt the mobile device was not the best way to do this:

*‘I know they’re expensive telephones and a simple pedometer at a fiver would have measured the number of paces I’d done’. (CHF 103)*

The view that the phone was an over-complicated solution was described as due to short battery life, bulkiness and the technical problems encountered:

*It might have been simple if you just had a simple pedometer… some days it hasn’t recorded anything at all’. (CHF107)*

Goal-setting was also supported as potentially motivational:

*‘I think it is quite easy to become idle. So if you have something that will drive you on a bit it’s good’. (CHF102)*

Although walking was seen as an appropriate goal, it was thought to be weather dependent and greater flexibility in terms of the goal activity would have been appreciated:

*‘I don’t want to be walking around when it’s cold and windy and wet’. (CHF109)*

Reviewing goals was also thought to be important, although in some cases participants were unable to complete the review (rate the walk as too easy, just right or too hard):

*‘Tried it front facing, back facing, sideways, no matter which way I carry it, it self-grades my walk’ (CHF105)*

Regular feedback provided on the home computer was seen as useful for some, but others did not look at it:

*‘That’s a good one where you can look at where you walk where you can see the bar graphs and where you can see how much you increased your walk’. (CHF109)*

*‘I didn’t look at them because I knew mentally and physically what I’d achieved’. (CHF104)*

In some cases the above mechanisms (M7) were perceived as having an effect on walking behaviour:

‘*Obviously when it tells you that you have to walk 13 minutes, well I walked 13 minutes… when it stopped working I didn’t go walking anymore’. (CHF109)*

However, one participant thought this would not result in long-term behaviour change (O7):

*‘I think people might drift back to their old ways’. (CHF106)*

In conclusion, as hypothesised; providing information did increase the knowledge level of people with lower levels of knowledge at the outset; technical difficulties had the hypothesised effect on ratings of usability, but did not affect actual use as originally envisaged; and the behaviour change strategies used to increase physical activity were perceived as potentially useful, although concerns were raised by some that walking as an activity, and the hardware used, might not be the most appropriate way to achieve this outcome.

### New CMO development

The results reported above have identified new CMOs to investigate, and indicate that the initial CMOs should be revised or rejected to provide a better description of how the PSMS for CHF might work, for whom and in what circumstances. Table 3 indicates the revised CMOs, and the emergent CMOs identified through this research.

## Discussion

The PSMS for CHF was evaluated according to a realist methodology [10,23]. The context of this study included individuals with congestive heart failure who had high levels of technology, symptom, and heart failure knowledge; who were satisfied with the relatively low-level involvement health care professionals had in their care; who recognised the need for changing behaviour as part of their ongoing self-management; and were relatively physically active at the outset. The PSMS was provided in the home, with minimal technical support. The mechanisms and outcomes supported in this context were that using technology to supply heart failure related information has the potential to increase heart failure related knowledge in those with lower baseline levels. In addition, using self-regulatory behaviour change techniques (self-monitoring, goal-setting, reviewing goals and receiving feedback) to encourage a daily walk could promote and support changes in walking behaviour. Although the predicted changes in physical fitness were not supported, several of the participants did report losing weight. Suggestions were made to improve these mechanisms, for example: simplifying the method of self-monitoring; ensuring goals are practical in terms of weather and abilities; ensuring the user has full control when reviewing goals; and ensuring feedback is accurate to reduce frustration. These mechanisms are likely to be strengthened by improvements in the usability and functioning of the system.

Realist evaluation is becoming more popular in health services research, however there are still some problems with how exactly to define a context or a mechanism [15] and whether these are mutually exclusive categories, or whether there could be movement between categories depending on how the outcome is affected [30]. It is important in future research to emphasise the cyclical nature of realist evaluation. The evidence collected using this method generates new hypotheses and revises existing ones. In this way, each iteration provides more information about what works, for whom, in what circumstance and how.

### External validity and limitations

The context of use for the study cannot be generalised to people with heart failure as a whole. Contextual factors such as: a wide range of technological abilities; low levels of activity; and poor awareness of symptoms leading to exacerbations, were identified as important when developing the CMO hypotheses, but were not represented in the sample recruited to the deployment phase. As this study was about the use of technology that included physical activity components, it is likely that the study was of more interest to those with an existing interest in technology and who are fairly active. We believe this has resulted in an unrepresentative sample, meaning certain contextual factors were not applicable. However, when developing the CMO hypotheses we may have underestimated the level of technology use of people with CHF. Recent figures from the Office of National Statistics show that in the UK 85.5% of adults aged 55–64, 67.4% of those 65–75 and 32.6% of those aged 75 or over had used the internet [31].

The act of participating in a research project is also a contextual factor that might have had an effect on outcomes such as system engagement. This context would not exist when evaluating service change, so there has been little investigation of it in terms of how it affects realist evaluations. It was hypothesised that the system would need to be reliable and fully functioning to result in engagement. However, while the technological problems experienced resulted in frustration for the participants, the number of physiological readings received was high. This indicates that interest and engagement with the system was not dependent on it being reliable and fully functioning. One possible explanation is that, in the context of participating in a research study, the experimental nature of the technology is clearly explained, and participants have made a commitment (in the form of completing informed consent) to use the technology, so the engagement seen here could have been influenced by the context of taking part in a research study. Following further development, if the PSMS is introduced to potential participants as a service change rather than a research project, and evaluated by people not directly involved in the development of the system, this may help reduce the potential social desirability bias.

In the TELER© analysis, there was a poorer response to questions where the negative response was correct (reversed statements, question 8–14 in Additional file 1: Appendix 2), and participants indicated in the interviews that these items were harder to understand. Presenting statements where both the positive and negative response is correct is important for good questionnaire design, however in the future perhaps the reversed statements could be counterbalanced to ensure this does not influence the change in knowledge scores.

Addressing the technological problems and exploring wider contexts of use could be explored in future iterations of the development, feasibility, and evaluation cycles. Potentially, purposive sampling of individuals with lower levels of activity, knowledge and technology experience could be used in future deployments.

In the future it may be possible to incorporate a self-management system such as the SMART PSMS into a telehealth system and transfer information to health care professionals, or into an electronic health record. This would be of particular relevance to those more at risk of exacerbations and would allow health care professionals to support and encourage the self-management of CHF in a more personalised way, whilst remotely monitoring health state in order to detect the signs of a forthcoming exacerbation.

The use of realist evaluation to inform future development of self-management technology may represent a more cost-effective way to identify unsupported hypotheses before wider adoption of technology by the health service. Identifying what works, for whom, in what circumstances might allow targeted delivery to those most likely to benefit, as well as informing continued development of alternative systems or approaches for those who are not likely to benefit.

## Conclusions

The CHF PSMS increased heart failure related knowledge in those with the lowest baseline scores and showed evidence of encouraging daily walking behaviour through goal-setting, self-monitoring and providing feedback in a sample of people with CHF who were stable, knowledgeable and active. The importance of behaviour change as part of self-managing CHF was recognised by this group; and despite encountering technological problems, the sample remained engaged and reported positively that technology may be an appropriate solution. Following stabilisation of the system, further testing of these mechanisms and outcomes in a range of contexts is needed to further develop this technology.

## Additional file

Additional file 1:
**Further details.** Contains the topics explored in the post-deployment interview and the health questions to measure change in knowledge.

## Abbreviations

CHF: Congestive Heart Failure
CMO: Context, Mechanism, Outcome
NHS: National Health Service
NICE: National Institute for Health and Care Excellence
PSMS: Personalised Self-Management System
SMART: Self-Management supported by Assistive, Rehabilitation and Telehealth technology
