# Supplementary material for: The SMART personalised self-management system for congestive heart failure: results of a realist evaluation
Source: BMC Med Inform Decis Mak. 2014 Nov 25;14:109. doi: 10.1186/s12911-014-0109-3 (PMC4246999; doi:10.1186/s12911-014-0109-3)
Supplement: Additional file 1: — Further details. Contains the topics explored in the post-deployment interview and the health questions to measure change in knowledge. [file 12911_2014_109_MOESM1_ESM.pdf]

Additional File 1

APPENDIX 1: *Topics explored during post deployment interviews to support or negate the conjectured CMOCs*

Topic areas explored during post deployment interviews for verification/falsification of conjectured CMOCs	*Associated Mechanism*
Potential users of the system have been involved in its design and development. We believed this would promote self-management in a home based setting because the system is a user-friendly (touch screen) piece of technology.	M1: Systems that have technical problems can result in low usability, and poor engagement. Therefore the system will be ‘glitch free’ and fully functioning.
As above.	M2: User-centred design process undertaken to identify a touch screen system with simple instructions designed to be operated by those with little or no computer knowledge
Doing too many things on the days you are feeling well could leave you with less energy on subsequent days. We believed the system would provide you with information (feedback) on your activities to allow you to see how much you were doing and help you to pace yourself accordingly.	M3. Pacing is taught by the system by providing feedback on activity, and showing users weekly plans, highlighting instances of over activity.
People with heart failure often live more sedentary lives and have been forced to curtail their hobbies and interests. Improving physical fitness is important in heart failure self-management. We believed that undertaking a daily walk would improve your overall fitness and give you more confidence to perform physical tasks in your day to day lives.	M4. Walking intervention to increase physical fitness
The system allowed you to monitor your blood pressure, weight and heart failure related symptoms (breathlessness, swelling, fatigue) on a daily basis remote from a health professional. We believed that this	M5. Increasing awareness of blood pressure, weight and symptoms through self-monitoring and tailored feedback provision

would enable you to be more aware of changes in your condition and improve your self-management skills	
Learning more about your condition is an important component of heart failure self-management. Providing this information is an element of your heart failure nurse’s role. We believed that providing you with heart failure specific information would increase your knowledge of your condition and lead to more effective self-management.	M6. Information and advice section contains educational material and quizzes, feedback from this and other sections should increase awareness.
You were asked to choose a goal to work towards (something you would like to do more of). We believed that this would make you more likely to use the system as it would be personalised to your lifestyle.	M7. The SMART2 system incorporates the following behaviour change techniques: 1. Self-monitoring of symptoms; 2. Setting and reviewing goals related to users’ lifestyle; 3. Providing regular feedback on performance

APPENDIX 2: *Health Questions to measure Change in Knowledge*

Number	Statement Text	Agree	Disagree
1	The heart is a muscular organ that acts like a pump to send blood round your body		
2	A common symptom of heart failure is shortness of breath		
3	A common symptom of heart failure is feeling tired		
4	A common symptom of heart failure is swelling (water retention)		
5	If you have heart failure you should try to include some form of physical activity into your lifestyle		
6	To help prevent heart failure you should try to stop smoking		
7	When your blood pressure is too high your heart has to work harder to pump blood around your body		
8	People can only get heart failure if they have had a heart attack		
9	Heart failure causes people to feel tired only after strenuous exercise		
10	If you have heart failure you should eat processed foods such as tinned soups		
11	Heart failure can be controlled by bed rest		
12	Heart failure can be controlled by increasing the amount of fluid you drink		

13	If you have heart failure you should eat a low carbohydrate diet		
14	If you miss a dose of medication it is ok to take a double dose		
15	Restricting your salt intake can help you in the management of your condition		
	Correct answer to the statement indicated by shading		
